# Inhaled nitric oxide in patients with acute respiratory distress syndrome caused by COVID-19: treatment modalities, clinical response, and outcomes

**DOI:** 10.1186/s13613-023-01150-9

**Published:** 2023-06-27

**Authors:** Armand Mekontso Dessap, Laurent Papazian, Manuella Schaller, Saad Nseir, Bruno Megarbane, Luc Haudebourg, Jean-François Timsit, Jean-Louis Teboul, Khaldoun Kuteifan, Marc Gainnier, Michel Slama, Patrick Houeto, Laurent Lecourt, Alain Mercat, Antoine Vieillard-Baron

**Affiliations:** 1 Service de Médecine Intensive Réanimation, Hôpitaux Universitaires Henri-Mondor, AP-HP, 94010 Créteil, France; 2 CARMAS research group, Univ Paris Est Créteil, Faculté de Santé, 94010 Créteil, France; 3 IMRB, INSERM, Univ Paris Est Créteil, 94010 Créteil, France; 4 Médecine Intensive Réanimation, Centre d’Etudes et de Recherches sur les Services de Santé et Qualité de vie EA 3279, Hôpital Nord, Assistance Publique–Hôpitaux de Marseille, Aix-Marseille University, 13015 Marseille, France; 5 Air Liquide Sante International, 92220 Bagneux, France; 6 Department of Intensive Care Medicine, Critical Care Center, CHU of Lille, 59000 Lille, France; 7 Department of Medical and Toxicological Critical Care, Lariboisière Hospital, INSERM MURS-1144, University of Paris, AP-HP, 2 Rue Ambroise Paré, 75010 Paris, France; 8 Service de Pneumologie et Réanimation Médicale du Département R3S, Groupe Hospitalier Pitié-Salpêtrière Charles Foix, AP-HP, Paris, France; 9 Medical and Infectious Diseases Intensive Care Unit, Bichat-Claude Hospital and U1137, IAME Université Paris-Cité, AP-HP, 75018 Paris, France; 10 Service de Medecine Intensive-Reanimation, Hôpital de Bicêtre, AP-HP, 78, rue du Général Leclerc, 94270 Le Kremlin-Bicêtre, France; 11 Service de Réanimation Médicale, Groupe Hospitalier de la Région Mulhouse Sud Alsace, 68100 Mulhouse, France; 12 Réanimation des Urgences, Hôpital de La Timone, Assistance Publique des Hôpitaux de Marseille, Marseille, France; 13 Medical Intensive Care Unit, CHU Sud Amiens, Amiens, France; 14 Department of Intensive Care, University of Angers, Angers, France; 15 Medical Intensive Care Unit, Ambroise Paré Hospital, AP-HP, Boulogne-Billancourt, France; 16 Inserm UMR 1018, Équipe 5, CESP, Villejuif, France

**Keywords:** COVID-19, Acute respiratory distress syndrome, Inhaled nitric oxide, Refractory hypoxaemia, ECMO

## Abstract

**Background:**

Inhaled nitric oxide (iNO) has been widely used in patients with COVID-19-related acute respiratory distress syndrome (C-ARDS), though its physiological effects and outcome are debated in this setting. The objective of this cohort study was to describe the modalities of iNO use, clinical response, and outcomes in a large cohort of C-ARDS patients.

**Methods:**

Multicentre, retrospective cohort study conducted in France.

**Results:**

From end February to December 2020, 300 patients (22.3% female) were included, 84.5% were overweight and 69.0% had at least one comorbidity. At ICU admission, their median (IQR) age, SAPS II, and SOFA score were 66 (57–72) years, 37 (29–48), and 5 (3–8), respectively. Patients were all ventilated according to a protective ventilation strategy, and 68% were prone positioned before iNO initiation. At iNO initiation, 2%, 37%, and 61% of patients had mild, moderate, and severe ARDS, respectively. The median duration of iNO treatment was 2.8 (1.1–5.5) days with a median dosage of 10 (7–13) ppm at initiation. Responders (PaO_2_/FiO_2_ ratio improving by 20% or more) represented 45.7% of patients at 6 h from iNO initiation. The severity of ARDS was the only predictive factor associated with iNO response. Among all evaluable patients, the crude mortality was not significantly different between responders at 6 h and their counterparts. Of the 62 patients with refractory ARDS (who fulfilled extracorporeal membrane oxygenation criteria before iNO initiation), 32 (51.6%) no longer fulfilled these criteria after 6 h of iNO. The latter showed significantly lower mortality than the other half (who remained ECMO eligible), including after confounder adjustment (adjusted OR: 0.23, 95% CI 0.06, 0.89, *p* = 0.03).

**Conclusions:**

Our study reports the benefits of iNO in improving arterial oxygenation in C-ARDS patients. This improvement seems more relevant in the most severe cases. In patients with ECMO criteria, an iNO-driven improvement in gas exchange was associated with better survival. These results must be confirmed in well-designed prospective studies.

**Supplementary Information:**

The online version contains supplementary material available at 10.1186/s13613-023-01150-9.

## Background

Several scientific societies have issued recommendations on the use of inhaled nitric oxide (iNO) in acute respiratory distress syndrome (ARDS). The guidelines are against the routine use of iNO, since there is no evidence of survival improvement [1], but suggest it may be considered in patients who remain severely hypoxemic despite optimal ventilation and other rescue strategies. This position is documented in the latest guidelines published by the French Intensive Care Society [2]*.*

Despite the paucity of clinical evidence, numerous publications suggest considering iNO in the management of refractory hypoxemia in patients with ARDS caused by COVID-19 (C-ARDS) [3–6]. Some theoretically beneficial properties of iNO, i.e., antiviral [7, 8], anti-inflammatory, and antithrombotic have also spurred its use in this setting. Published studies report conflicting results on the effect of iNO on blood oxygenation in C-ARDS. In addition, the role of iNO in the management of severe hypoxemia caused by COVID-19 remains largely debated [9–13], and most published cohorts have a limited sample size, of 34 (10–122) in a recent meta-analysis [14]. The mechanisms of hypoxemia in C-ARDS are complex and the role of hypoxic vasoconstriction is a controversial subject [15].

We hypothesized that iNO may improve oxygenation in a significant number of patients with C-ARDS, and consequently may influence their outcome. The objectives of our cohort study were to describe the use of iNO in a large cohort of C-ARDS patients, to report patient’s response, and to investigate the outcomes of this treatment.

## Methods

This is a retrospective cohort study conducted in 12 intensive care units (ICUs) in France. The study included all consecutive patients of over 18 years admitted to participating ICUs between February 25 and December 31, 2020, who were treated with iNO for at least 1 h for laboratory-confirmed COVID-19 ARDS [16] and did not object to the use of their personal health data. The study protocol was submitted to Health Data Hub, the regulatory body in charge of validating projects carried out on existing databases in France [17], in April 2021.

Data were recorded on patients’ demographic characteristics and comorbidities; clinical and laboratory findings at different timepoints (ICU admission, intubation, diagnosis of ARDS, initiation of iNO treatment, during treatment, after discontinuation); modalities of iNO administration (type of delivery device, monitoring, dosage); severity and organ failure according to Simplified Acute Physiology Score (SAPS) II [18] and Sepsis-related Organ Failure Assessment (SOFA) score [19]; use of prone position, renal replacement therapy or extracorporeal membrane oxygenation (ECMO); ICU and hospital lengths of stay; and in-ICU, and in-hospital mortality. Safety assessment focused on the need for renal replacement therapy and methaemoglobin level during iNO treatment.

Patient management followed a lung protective approach [2] in accordance with the recommendations of the French Society of Intensive Care Medicine for COVID-19 [20].

The response to iNO was defined as positive if the ratio of partial oxygen pressure in arterial blood to fraction of inspired oxygen (PaO_2_/FiO_2_) relatively changed by 20% or more between the last arterial blood gas (ABG) sampled before iNO initiation and a subsequent ABG sampled after iNO initiation [21]. Response to iNO was assessed at different timepoints: within 6 h (H6), within 1 day (H24), or at any time during iNO administration. The two ABG samples considered to assess iNO response were drawn in the same position, i.e., either supine or prone.

A subgroup analysis was conducted in patients who had, at time of iNO initiation, refractory ARDS as defined by the fulfilment of ECMO criteria as per EOLIA study [22], i.e., a PaO_2_/FiO_2_ ratio < 80 mmHg or PaCO_2_ ≥ 60 mmHg and pH < 7.25. This subgroup was stratified into those whose condition improved within 6 h of initiation of iNO treatment in a way that ECMO was no longer indicated as per EOLIA criteria, and those who still fulfilled these criteria (unchanged condition).

The safety analysis focussed on a circumscribed set of parameters usually encountered as possible adverse reactions to iNO treatment, namely, impairment of kidney function requiring renal replacement therapy and elevated methaemoglobin level.

## Statistical analysis

For sample size calculation, we considered previous reports suggesting a positive response rate to iNO ranging from 25% to 65% [11, 23]. We assumed that we would reach a response rate of at least 50%. To reach a precision of 6% with 95% confidence (alpha risk of 5%) around the target response rate, a sample size of 250 patients was needed. Categorical data are described as frequencies and percentages of subjects in each category. Percentages were calculated based on non-missing observations. Continuous variables are described as medians and interquartile ranges. Some variables were compared using chi-square or Fisher's exact test for categorical variables, and Student's t or Wilcoxon's rank-sum test for continuous variables, as appropriate. The association between iNO response and in-hospital death in patients with refractory ARDS was assessed using a multivariable logistic regression. Only factors measured before iNO initiation were selected. Considering the limited sample size of the subgroup, we selected plausible confounding factors that were hypothesized as being the most relevant based on experts’ knowledge on the field [24] and that were not related to each other. To quantify this association, crude odds ratio and adjusted odds ratio with their associated 95% confidence intervals are presented. A *p* value of < 0.05 was considered statistically significant. All statistical analyses were performed using SAS, version 9.4 (SAS Institute).

## Results

### Patients

**Fig. 1:**
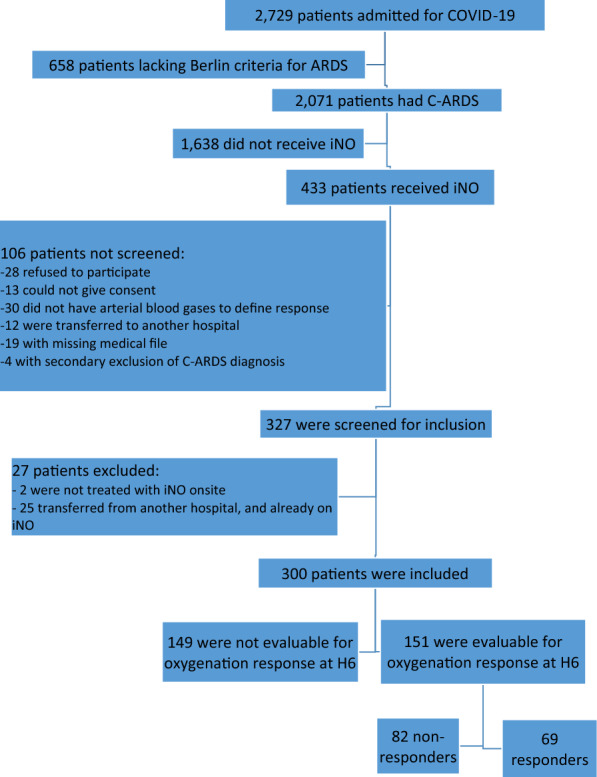
Study flow chart

Over the study period and in its 12 participating centers, 2729 patients were admitted for COVID-19, of whom 2071 had ARDS, including 433(20.9%) treated with iNO. Of the latter, 327 were screened for eligibility and 300 fulfilled the selection criteria of this study (Fig. 1). The study population were predominantly male, over 60 years, with overweight and other comorbidities (Additional file 1: Table S1). Typically, patients were admitted to ICU about 1 week from the onset of COVID, and were intubated on admission day (Additional file 1: Table S1). Ventilation parameters and condition-assessment scores at the time of iNO initiation indicated severely compromised respiratory function (median PaO_2_/FiO_2_ ratio < 100 mmHg, and most patients put on prone position between intubation and iNO initiation) and hemodynamic status (median cardiovascular SOFA score of 3) (Additional file 1: Table S2). Altogether, 105 patients (35%) were discharged alive from hospital and their median length of stay in ICU and in hospital were 38 (26–51) and 49 (36–73) days, respectively.

### iNO administration

iNO therapy was initiated after a median (IQR) of 7 (3–12) days following ICU admission and 4 (1–10) days after intubation. iNO was delivered at a median dosage of 10 (7–13) ppm for a median duration of 3 (1–6) days, mainly in a continuous delivery mode (Additional file 1: Table S2). The reasons for stopping iNO were death (25.3%), weaning after a treatment deemed clinically “successful” (37.5%), or “failure” (35.8%) by the intensivist, and toxicity (1.4%). In 26 (8.7%) patients, it was decided to resume iNO treatment immediately after an attempt to stop (suggesting a rebound effect) and iNO treatment was resumed after an interruption of 48 h or more in 51 patients, for an additional median duration of 86 (26–158) h. A majority of patients (*n* = 156/269 with available data, 58.0%) were proned at least once in the 24 h following iNO initiation. Almitrine was concomitantly administered with iNO in 56 (18.7%) patients.

### Oxygenation response to iNO

**Fig. 2:**
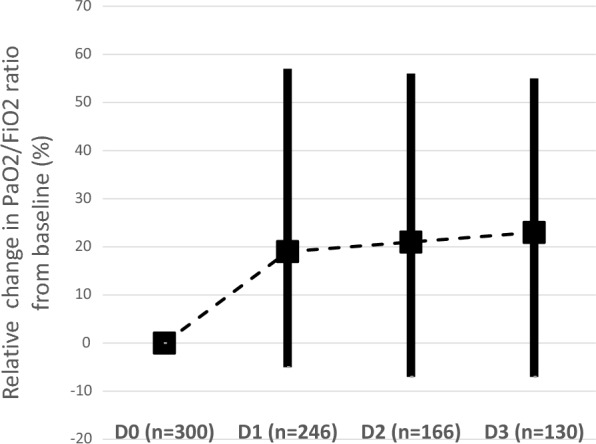
Median relative change from baseline in ratio of partial oxygen pressure in arterial blood to fraction of inspired oxygen. D0, D1, D2, and D3 denote baseline, the first, second, and third day of inhaled nitric oxide administration, respectively; boxes represent median values and bars represent interquartile ranges; PaO_2_, partial oxygen pressure in arterial blood; FiO_2_, fraction of inspired oxygen

The median (IQR) interval between iNO initiation and ABG measurement documenting responsiveness was 7 (2–23) h. A positive oxygenation response to iNO was observed in 46%, 57%, and 70% of patients evaluable at H6 (n = 151), H24 (*n* = 246), and at any time during iNO administration (*n* = 256), respectively (Additional file 1: Table S3). The percentage of patients classified as severe ARDS in supine position decreased from 60.0% (135/225) down to 48.2% (110/228) within 24 h of iNO initiation. Oxygenation improvement was sustained throughout the course of iNO treatment (Fig. 2).

The only significant differences that emerged between the 151 patients whose data allowed us to evaluate their oxygenation response to iNO within 6 h of initiation and those whose collected data did not allow this analysis included more prone position sessions, higher values of respiratory rate and plateau pressure, and higher mortality in the latter group (Additional file 1: Tables S1 and S2). Evaluable patients received iNO for a longer duration, but with less nitric dioxide monitoring (Additional file 1: Table S2).

**Fig. 3:**
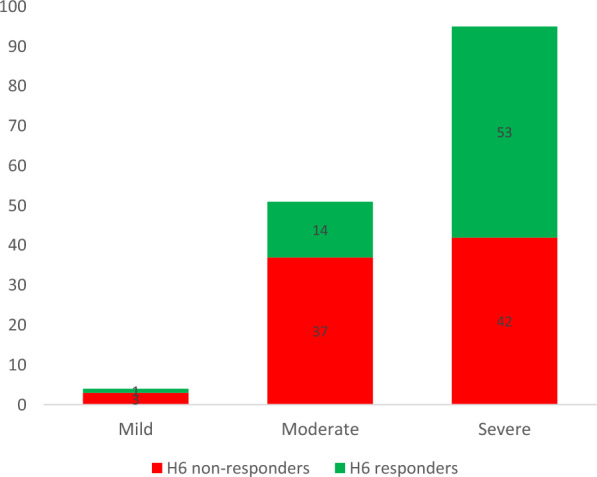
Number of patients with mild, moderate and severe acute respiratory distress syndrome (classified before inhaled nitric oxide initiation) according to their oxygenation response within 6 h. H6 denotes 6 h

The median (IQR) PaO_2_/FiO_2_ before iNO initiation was lower in H6 responders as compared with non-responders, 79 (61–99) vs. 109 (81–136) mmHg, *p* < 0.001. Additional file 1: Table S4 reports absolute and relative changes in ABG variables assessed within 6 h of iNO treatment. ARDS severity was the only factor associated with oxygenation response at H6, with higher response rates observed in severe ARDS patients (Table 1, Fig. 3). In-hospital mortality was 52.2% (36/69) in H6 responders vs. 64.6% (53/82) in H6 non-responders, *p* = 0.12 (Table 2).

**Table 1:** Respiratory parameters and modalities of inhaled nitric oxide administration in patients with COVID-19-related acute respiratory distress syndrome receiving inhaled nitric oxide, according to the oxygenation response within 6 h

	AD^a^	All patients *N* = 151	Oxygenation response at H6		*p* value
			Yes *N* = 69	No *N* = 82	
ARDS severity at diagnosis	111				0.948
Mild		5 (5%)	2 (4%)	3 (5%)	
Moderate		38 (34%)	18 (33%)	20 (35%)	
Severe		68 (61%)	34 (63%)	34 (60%)	
ARDS severity within 6 h before iNO initiation	150				0.002
Mild		4 (3%)	1 (1%)	3 (4%)	
Moderate		51 (34%)	14 (21%)	37 (45%)	
Severe		95 (63%)	53 (78%)	42 (51%)	
ABG before iNO initiation, prone position					
PaO_2_/FiO_2_	44	79 (68–95)	79 (68–95)	80 (69–95)	0.953
PaO_2_, mmHg	44	71 (63–80)	71 (64–79)	71 (60–80)	0.879
FiO_2_, %	44	100(80–100)	100 (80–100)	100(80–100)	0.625
PaCO_2_, mmHg	44	48 (43–60)	46 (42–58)	49 (44–66)	0.250
pH	44	7.32 (7.24–7.40)	7.32 (7.24–7.41)	7.35 (7.24–7.39)	0.787
ABG before iNO initiation, supine position					
PaO_2_/FiO_2_	129	90 (71–122)	79 (61–99)	109 (81–136)	< 0.001
PaO_2_, mmHg	129	72 (62–87)	68 (57–78)	76 (67–98)	0.001
FiO_2_, %	129	90 (70–100)	100 (71–100)	80 (70–100)	0.035
PaCO_2_, mmHg	129	49 (40–55)	48 (37–55)	49 (41–56)	0.270
pH	129	7.36 (7.28–7.42)	7.38 (7.28–7.42)	7.35 (7.28–7.41)	0.421
ABG before iNO initiation					
PaO_2_/FiO_2_	151	86 (71–119)	79 (64–97)	98 (74–131)	< 0.001
PaO_2_, mmHg	151	72 (64–85)	71 (61–79)	74 (67–90)	0.018
FiO_2_, %	151	90 (70–100)	100 (80–100)	80 (70–100)	0.009
PaCO_2_, mmHg	151	49 (41–58)	48 (39–57)	49 (43–58)	0.246
pH	151	7.35 (7.27–7.41)	7.34 (7.28–7.42)	7.35 (7.27–7.41)	0.662
Time interval between					
Disease onset and ICU admission, days	147	8 (6–10)	7 (6–10)	8 (6–10)	0.589
ICU admission and ARDS diagnosis, days	141	0 (0–2)	0 (0–2)	0 (0–2)	0.819
ARDS diagnosis and intubation, days	148	0 (0–1)	0 (0–1)	0 (0–1)	0.437
Intubation and iNO initiation, days	151	4 (1–9)	3 (0–8)	5 (1–9)	0.140
ICU admission and iNO initiation, days	151	7 (3–12)	7 (2–11)	7 (4–12)	0.489
Ventilatory status					
Intubated within 24 h after ICU admission	148	78 (53%)	38 (56%)	40 (50%)	0.475
Intubated after iNO initiation	151	1 (0.7%)	1 (1–0.4%)	0 (0.0%)	0.457
Prone position before iNO initiation	151	99 (65.6%)	42 (60.9%)	57 (69.5%)	0.266
Number of prone position sessions before iNO initiation	97	2 (1–3)	1 (1–3)	2 (1–3)	0.502
Almitrine at time of iNO administration	151	31 (20.5%)	16 (23.2%)	15 (18.3%)	0.4581
Ventilation parameters at iNO initiation					
Respiratory rate, cpm	145	28 (24–30)	27 (24;30)	29 (24;32)	0.247
Plateau pressure, cmH_2_O	107	28 (25–31)	28 (25–31)	27 (22–30)	0.115
FiO_2_ (%)	145	98 (70–100)	99 (80–100)	90 (70–100)	0.333
Positive end expiratory pressure, cmH_2_O	143	12 (9–14)	12 (10–14)	12 (8–14)	0.528
Tidal volume, mL/kg PBW	132	6.1 (5.8–6.8)	6.2 (5.5–7.1)	6.1 (5.9–6.5)	0.578
iNO modalities					
iNO dosage at initiation, ppm	140	10 (7–13)	10 (7–13)	10 (6–13)	0.905
Monitoring of nitric dioxide	151	33 (22%)	14 (20%)	19 (23%)	0.670
Duration of iNO administration, days	149	3.1 (1.4–6.5)	4.2 (1.8,7.0)	2.7 (1.2,5.3)	0.073
Type of ventilation device	121				0.542
Continuous delivery (Minikinox-type)		69 (57.0%)	34 (61.8%)	35 (53.0%)	
Sequential mode (Optikinox)		32 (26.4%)	12 (21.8%)	20 (30.3%)	
Synchronized with ventilators		20 (16.5%)	9 (16.4%)	11 (16.7%)	

^a^Denotes available data; H6 denotes 6 h; iNO, inhaled nitric oxide; ICU, intensive care unit; PaO_2_, partial oxygen pressure in arterial blood; FiO_2_, fraction of inspired oxygen; PaCO_2_, partial pressure of carbon dioxide

**Table 2:** Clinical characteristics of patients with COVID-19-related acute respiratory distress syndrome receiving inhaled nitric oxide, according to the oxygenation response within 6 h

	AD^a^	All patients *N* = 151	Oxygenation response at H6		*p* value
			Yes *N* = 69	No *N* = 82	
Patient’s characteristics					
Age, years	151	65 (56–72)	66 (53–72)	65 (56–72)	0.929
Women, *n* (%)	151	31 (20.5%)	11 (16%)	20 (24%)	0.200
Body Mass Index ≥ 30 kg/m^2^	144	72 (50%)	30 (46%)	42 (54%)	0.316
At least one comorbidity	151	110 (72.8%)	48 (69.6%)	62 (75.6%)	0.405
Treated hypertension		68 (45.0%)	31 (44.9%)	37 (45.1%)	0.981
Diabetes		60 (39.7%)	22 (31.9%)	38 (46.3%)	0.071
COPD		11 (7.3%)	5 (7.2%)	6 (7.3%)	0.987
Immunodeficiency		11 (7.3%)	5 (7.2%)	6 (7.3%)	0.987
Condition at ICU admission					
SAPS II score	114	35 (29–45)	40 (29–51)	34 (28–39)	0.054
SOFA score	119	4 (3–7)	4 (3–8)	5 (4–7)	0.703
Cardiovascular SOFA score	109	1 (0–3)	0 (0–3)	2 (0–3)	0.601
Condition within 6 h before iNO initiation					
SOFA score	114	8 (5–12)	8 (5–12)	8 (5–13)	0.798
Renal SOFA score	94	0 (0–2)	0 (0–1)	0 (0–2)	0.326
Cardiovascular SOFA score	120	3 (0–4)	3 (0–4)	1 (0–4)	0.455
Biological parameters before iNO initiation					
Creatinine, µmol/L	144	90 (62–187)	80 (62–140)	107 (62–236)	0.184
Platelets, G/L	102	241 (196–320)	231 (196–297)	249 (188–368)	0.410
Outcomes					
ICU length of stay in survivors, days	62	38 (26–51)	38 (27–44)	41 (22–55)	0.871
Hospital length of stay in survivors, days	59	49 (39–73)	48 (41–79)	53 (36–70)	0.538
ICU mortality	151	86 (57.0%)	35 (50.7%)	51 (62.2%)	0.156
Hospital mortality	151	89 (58.9%)	36 (52.2%)	53 (64.6%)	0.121

^a^Denotes available data; H6 denotes 6 h; iNO, inhaled nitric oxide; ICU, intensive care unit; SAPS, simplified acute physiology score; SOFA, Sequential Organ Failure Assessment; COPD, chronic obstructive pulmonary disease

### Subgroup of refractory ARDS

Refractory ARDS subgroup comprised 62 patients who fulfilled EOLIA criteria for ECMO at the time of iNO initiation. Within 6 h of initiating iNO treatment, nearly half of these patients were no longer eligible for ECMO (regression of ECMO eligibility criteria). The latter showed lower mortality rate than the other half (who remained ECMO eligible) [18/30 (60.0%) vs. 27/32 (84.4%), *p* = 0.032, crude OR: 0.246 [95%CI 0.069; 0.873]; *p* = 0.03], even after adjustment for age, enrolment period (pandemic wave), time between intubation and iNO initiation, and SOFA score before iNO initiation (adjusted OR: 0.231 [0.060; 0.890], *p* = 0.03). Of the total 62 initially eligible patients, only 19/62 (30.6%) eventually received ECMO, in a pandemic context with major resource limitation, and with no difference between patients with regression of ECMO eligibility criteria under iNO [9/30 (30.0%)] and those who remained ECMO eligible [10/32 (31,3%)].

### Safety

At iNO initiation, 12.0% (36/299, one missing data) of patients required renal replacement therapy. During iNO treatment, renal replacement therapy was weaned in 9 (4.9%) patients and initiated in 43 (23.5%) patients. No marked increase in methaemoglobin level was documented apart from one patient whose level rose above the 5% threshold.

## Discussion

We herein report the largest study conducted in patients treated with iNO for C-ARDS. Our main findings are: (i) iNO improved oxygenation in half of the patients at 6 h of treatment, and ARDS severity was the main factor associated with oxygenation improvement; (ii) Half of patients with refractory ARDS fulfilling ECMO criteria were no longer eligible for ECMO after iNO administration, and this loss of eligibility was associated with a lower mortality.iNO was used in one-fifth of patients with C-ARDS in our cohort, which is consistent with other series in patients with C-ARDS. During the pandemic, this treatment was administered to 425/2224 (19%) patients in France [25]. In contrast, only 7.7% of patients with non COVID-19-related ARDS were treated with iNO in the LUNG SAFE study [26]. The higher use of iNO in C-ARDS, as compared with classical ARDS, could be explained by the massive influx of patients which gave no time for the application of rescue (e.g., ECMO) therapies during the pandemic.

The effect of iNO on blood oxygenation was controversial in C-ARDS. For instance, preliminary small studies have reported no major effect on oxygenation improvement [13]. To the contrary, nearly half of our patients showed a significant improvement in oxygenation at H6 of iNO treatment, and the absolute change in PaO_2_/FiO_2_ ratio was similar to that found in patients with classical ARDS (median value of 15 mmHg) [1]. The significant improvement in responders may still suggest a role of hypoxic vasoconstriction, a role that is already widely disputed in C-ARDS. Some authors describe a loss of hypoxic pulmonary vasoconstriction in C-ARDS, unlike in classical ARDS, which may explain a higher lung shunt and hypoxemia [15]. Besides, autopsy [27] and CT-scan [28] studies have suggested a prominent role of pulmonary vascular occlusion and angiogenesis in C-ARDS, but one study found no evidence of major intrapulmonary anatomical shunt [29]. 

The oxygenation response was greater in the more hypoxemic patients, similar to that observed in patients with classical ARDS [1]. Using relative change in PaO_2_/FiO_2_ ratio to define response may bring along a detection bias, which we believe is unlikely given the similar change in absolute values of PaO_2_/FiO_2_ in responders of various severity degrees.

Many C-ARDS patients exhibit acute cor pulmonale [30]. Whether iNO can relieve acute cor pulmonale in this setting warrants further research as the role of pulmonary thrombosis seems more important in this situation, than it is in classical ARDS [31] and the efficacy of iNO is questionable in preliminary reports [32]. In this regard, iNO could worsen ventilation/perfusion mismatch [33], which was not observed in our study, albeit we were unable to systematically report pulmonary embolism screening in our patients.

In patients with refractory ARDS, as defined by fulfilment of ECMO criteria as per EOLIA study, half of them lost ECMO eligibility after iNO administration. This high rate is reasoned by the important role of refractory hypoxemia in ECMO criteria, and is supported by the association of oxygenation response to iNO with ARDS severity level. Only a minority of ECMO-eligible patients actually received ECMO, probably because the healthcare situation during the epidemic imposed local adaptation of ECMO criteria to fit the increased demand and the shortage of machines and staff. The better prognosis of iNO-treated patients who no longer fitted ECMO criteria, as compared with those who remained eligible, may reinforce the recommendation to test iNO before initiating ECMO [2, 22]. It may also suggest considering the assessment of pulmonary vascular dysfunction as an ECMO eligibility criterion [34]. Further studies are needed to assess whether systematic use of iNO in all ECMO-eligible patients may reduce the resort to ECMO and its associated morbidity.

Strengths of our study reside in its size, multicentric approach, and pragmatic design. Our study has several limitations. First, it was retrospective and observational with wide inclusion criteria and no control arm. We could not compare the evolution of oxygenation between exposed and non-exposed patients. For such, we rigorously collected data and mainly analysed patients with no missing data for iNO response. This explains why the response to iNO was only reported in half of the population. However, we do not report significant differences between patients evaluable for response and the others. Our cohort study also included all patients receiving iNO for ARDS whatever its severity. Our observation of residual use of iNO in mild ARDS is in accordance with the data from the Lung Safe study [26], where 3.4% of patients with mild ARDS received inhaled vasodilators. Second, our patients were treated during a pandemic period, which may limit the external validity of our results. Third, mortality in our cohort is higher than that found in the literature (ranging from 32% to 49% for moderate and severe COVID-related ARDS, respectively [25]). This higher rate of mortality may be related to the selection of the most hypoxemic patients to receive iNO on the clinical ground, especially in a period of shortage of some delivery devices. Some studies performed in the US showed an even higher level of mortality (78%) in patients with refractory hypoxaemia [35]. Fourth, a regression to the mean phenomenon cannot be excluded given the overall severity and high mortality of our cohort with no control group. However, our selection criteria were not based on ARDS severity. It is unlikely that patients with more severe hypoxemia were more likely to be NO responders as an artefact of our definition of NO response, because these patients had both the highest absolute and relative changes in PaO_2_/FiO_2_ (Additional file 1: Table S4). Finally, the group with refractory ARDS had a limited sample size. The multivariable logistic regression model run on this subgroup included less than 10 events per predictor variable (EPV). Peduzzi et al. [36] recommended a minimum of 10 EPV in regression model. However in our logistic regression model, the covariates are used for the purpose of controlling for confounding factors and not as predictors. In this context of analysis of causal influences in observational data, Vittinghoff et al. [37] suggest that this rule of thumb can be relaxed. One concern with low EPV is an increased risk of separation, i.e., that a covariate or a linear combination of covariates separates all events from all non-events leading to convergence issues of the iterative maximum likelihood estimation procedure. However, no such convergence issues arose in our analysis. In this small size subgroup, the odds ratio might have been overestimated and results should, therefore, be used for hypothesis generation and confirmed on a wider scale.

In conclusion, nearly half of C-ARDS showed improved oxygenation response to iNO, especially those with severe ARDS. In the subgroup of ECMO-eligible patients, those who lost eligibility status within 6 h of iNO treatment subsequently showed lower mortality. Further studies are needed to better scrutinize the role of systematic use of iNO in this subgroup of patients in the era of protective ventilation and prone positioning.

## Supplementary Information

**Additional file 1: Table S1.** Clinical characteristics of patients with COVID-19-related acute respiratory distress syndrome receiving inhaled nitric oxide in patients evaluable at 6 h following iNO and the others. **Table S2.** Respiratory parameters and modalities of inhaled nitric oxide administration in patients with COVID-19-related acute respiratory distress syndrome in patients evaluable at 6 h following iNO and the others. **Table S3.** Oxygenation response to inhaled nitric oxide therapy in patients with COVID-19-related acute respiratory distress syndrome. **Table S4.** Change in arterial blood gas variables within 6 h of inhaled nitric oxide therapy in patients with COVID-19-related mild, moderate and severe acute respiratory distress syndrome.

## Data Availability

The trial steering committee will work to make study data available on legitimate requests.
