# Supplementary material for: Inhaled nitric oxide in patients with acute respiratory distress syndrome caused by COVID-19: treatment modalities, clinical response, and outcomes
Source: Ann Intensive Care. 2023 Jun 27;13:57. doi: 10.1186/s13613-023-01150-9 (PMC10299982; doi:10.1186/s13613-023-01150-9)
Supplement: Supplementary file 1 — Additional file 1: Table S1. Clinical characteristics of patients with COVID-19-related acute respiratory distress syndrome receiving inhaled nitric oxide in patients evaluable at 6 h following iNO and the others. Table S2. Respiratory parameters and modalities of inhaled nitric oxide administration in patients with COVID-19-related acute respiratory distress syndrome in patients evaluable at 6 h following iNO and the others. Table S3. Oxygenation response to inhaled nitric oxide therapy in patients with COVID-19-related acute respiratory distress syndrome. Table S4. Change in arterial blood gas variables within 6 h of inhaled nitric oxide therapy in patients with COVID-19-related mild, moderate and severe acute respiratory distress syndrome. [file 13613_2023_1150_MOESM1_ESM.docx]

## Table S1. Clinical characteristics of patients with COVID-19-related acute respiratory distress syndrome receiving inhaled nitric oxide in patients evaluable at 6 hours following iNO and the others

|  |  |  | **Oxygenation response**  **at H6** | |  |
| --- | --- | --- | --- | --- | --- |
|  | **AD*** | **All patients**  **N=300** | **Not evaluable**  **N=149** | **Evaluable**  **N=151** | **P-**  **value** |
| **Patient’s characteristics** |  |  |  |  |  |
| Age, years | 300 | 66 (57-72) | 66 (59-72) | 65 (56-72) | 0.470 |
| Women, n (%) | 300 | 67 (22.3%) | 36 (24.2%) | 31 (20.5%) | 0.450 |
| Body Mass Index  ≥30kg/m^2^ | 284 | 139 (48.9%) | 67 (47.9%) | 72 (50%) | 0.718 |
| At least one comorbidity | 300 | 207 (69.0%) | 97 (65.1%) | 110 (72.8%) | 0.147 |
| Treated hypertension |  | 141 (47.0%) | 73 (49.0%) | 68 (45.0%) | 0.492 |
| Diabetes |  | 111 (37.0%) | 51 (34.2%) | 60 (39.7%) | 0.323 |
| COPD |  | 28 (9.3%) | 17 (11.4%) | 11 (7.3%) | 0.219 |
| Immunodeficiency |  | 18 (6.0%) | 7 (4.7%) | 11 (7.3%) | 0.346 |
| **Condition at ICU admission** | | | | | |
| SAPS II score | 222 | 37 (29 – 48) | 38 (31-50) | 35 (29-45) | 0.260 |
| SOFA score | 218 | 5 (3-8) | 5 (3-8) | 4 (3-7) | 0.854 |
| Cardiovascular SOFA score | 200 | 0 (0-3) | 0 (0-3) | 1 (0-3) | 0.221 |
| **Condition 6h before iNO initiation** | | | | | |
| SOFA score | 170 | 8 (5-11) | 8 (6-11) | 8 (5-12) | 0.344 |
| Renal SOFA score | 162 | 0 (0-2) | 0 (0-2) | 0 (0-2) | 0.845 |
| Cardiovascular SOFA score | 183 | 3 (0-4) | 3 (0-4) | 3 (0-4) | 0.446 |
| **Biological parameters before iNO initiation** | | | | | |
| Creatinine, µmol/L | 286 | 91 (63-170) | 91 (67-150) | 90 (62-187) | 0.775 |
| Platelets, G/L | 192 | 249(188– 333) | 257(183- 337) | 241(196-320) | 0.983 |
| **Outcomes** |  |  |  |  |  |
| ICU LOS in survivors, days | 105 | 38 (26 - 51) | 34 (26-51) | 38 (26 - 51) | 0.560 |
| Hospital LOS in survivors, days | 97 | 49 (36 - 73) | 43 (31 - 74) | 49 (39-73) | 0.297 |
| ICU mortality | 300 | 191 (63.7%) | 105 (70.5%) | 86 (57.0%) | 0.0149 |
| Hospital mortality | 300 | 195 (65.0%) | 106 (71.1%) | 89 (58.9%) | 0.0267 |

*Denotes available data; H6 denotes six hours; iNO, inhaled nitric oxide; ICU, intensive care unit; SAPS, simplified acute physiology score; SOFA, Sequential Organ Failure Assessment; LOS, length of stay; COPD, chronic obstructive pulmonary disease.

## Table S2. Respiratory parameters and modalities of inhaled nitric oxide administration in patients with COVID-19-related acute respiratory distress syndrome in patients evaluable at 6 hours following iNO and the others

|  |  |  | **Oxygenation response**  **at H6** | |  |
| --- | --- | --- | --- | --- | --- |
|  | **AD*** | **All patients**  **N=300** | **Not evaluable**  **N=149** | **Evaluable**  **N=151** | **P-**  **value** |
| **ARDS severity at diagnosis** | 214 |  |  |  | 0.505 |
| Mild |  | 11 (5%) | 6 (6%) | 5 (5%) |  |
| Moderate |  | 80 (37%) | 42 (41%) | 38 (34%) |  |
| Severe |  | 123 (58%) | 55 (53%) | 68 (61%) |  |
| **ARDS severity within 6h before**  **iNO initiation** | 278 |  |  |  | 0.315 |
| Mild |  | 5 (2%) | 1 (1%) | 4 (3%) |  |
| Moderate |  | 103 (37%) | 52 (40%) | 51 (34%) |  |
| Severe |  | 170 (61%) | 75 (59%) | 95 (63%) |  |
| **ABG before iNO initiation, prone position** | | | | | |
| PaO_2_/FiO_2_ | 92 | 84 (71-114) | 94 (73-131) | 79 (68-95) | 0.055 |
| PaO_2_, mmHg | 95 | 73 (64-92) | 80 (66-97) | 71 (63-80) | 0.050 |
| FiO_2_, % | 92 | 100 (80 – 100) | 90 (80-100) | 100 (80–100) | 0.514 |
| PaCO_2_, mmHg | 95 | 50 (44-58) | 51 (45-58) | 48 (43-60) | 0.499 |
| PH | 95 | 7.32 (7.25-7.40) | 7.32 (7.27-7.39) | 7.32 (7.24-7.40) | 0.970 |
| **ABG before iNO initiation, supine position** | | | | | |
| PaO_2_/FiO_2_ | 227 | 89 (66-120) | 87 (64-116) | 90 (71-122) | 0.182 |
| PaO_2_, mmHg | 232 | 71 (60-85) | 65 (57-83) | 72 (62-87) | 0.077 |
| FiO_2_, % | 230 | 90 ( 70-100) | 90  (75-100) | 90 (70-100) | 0.438 |
| PaCO_2_, mmHg | 235 | 49 (41-57) | 50 (42-58) | 49 (40-55) | 0.410 |
| PH | 235 | 7.35 (7.28-7.42) | 7.35 (7.30-7.40) | 7.36 (7.28-7.42) | 0.894 |
| **Time interval between** |  |  |  |  |  |
| Disease onset and ICU admission, days | 290 | 8 (6-11) | 8 (5-11) | 8 (6-10) | 0.517 |
| ICU admission and ARDS diagnosis, days | 275 | 0 (0-2) | 0 (0-1) | 0 (0-2) | 0.355 |
| ARDS diagnosis and intubation, days | 294 | 0 (0-1) | 0 (0-1) | 0 (0-1) | 0.424 |
| Intubation and iNO initiation, days | 297 | 4 (1-10) | 5 (1-11) | 4 (1-9) | 0.089 |
| ICU admission and iNO initiation, days | 298 | 7 (3-13) | 8 (3-13) | 7 (3-12) | 0.259 |
| **Ventilatory status** |  |  |  |  |  |
| Intubated within 24h after ICU admission | 292 | 161 (55%) | 83 (58%) | 78 (53%) | 0.396 |
| Intubated after iNO initiation | 298 | 2 (0.7%) | 1 (0.7%) | 1 (0.7%) | >0.99 |
| Prone position before iNO initiation# | 299 | 204 (68%) | 105 (71%) | 99 (65.6%) | 0.318 |
| Number of prone position sessions before iNO initiation | 201 | 2 (1-4) | 3 (1-4) | 2 (1-3) | 0.014 |
| Almitrine at time of iNO administration |  | 56 (18.7%) | 25 (16.8%) | 31 (20.5%) | 0.404 |
| **Ventilation parameters at iNO initiation** |  |  |  |  |  |
| Respiratory rate, cpm | 280 | 28 (24-32) | 30 (24-35) | 28 (24-30) | 0.035 |
| Plateau pressure, cm H_2_O | 201 | 28 (25-32) | 29 (27-33) | 28 (25-31) | 0.007 |
| FiO_2_ (%) | 279 | 100 (75-100) | 100 (80-100) | 98 (70-100) | 0.087 |
| Positive end expiratory pressure, cm H_2_O | 270 | 12 (8-14) | 12 (8-14) | 12 (9-14) | 0.910 |
| Tidal volume, mL/kg PBW | 250 | 6.2 (5.8-6.7) | 6.2 (5.7-6.6) | 6.1 (5.8-6.8) | 0.586 |
| **iNO modalities** |  |  |  |  |  |
| iNO dosage at initiation, ppm | 273 | 10 (7-13) | 10 (8-14) | 10 (7-13) | 0.377 |
| Monitoring of nitric dioxide | 298 | 80 (27%) | 47 (32%) | 33 (22%) | 0.049 |
| Duration of iNO administration, days | 291 | 2.8 (1.1-5.5) | 2.5 (0.9-4.6) | 3.1 (1.4-6.5) | 0.018 |
| Type of ventilation device | 240 |  |  |  | 0.358 |
| Continuous delivery (Minikinox-type) |  | 134 (55.8%) | 65 (54.6%) | 69 (57.0%) |  |
| Sequential mode (Optikinox) |  | 58 (24.2%) | 26 (21.8%) | 32 (26.4%) |  |
| Synchronized with ventilators |  | 48 (20.0%) | 28 (23.5%) | 20 (16.5%) |  |

*Denotes available data; H6 denotes six hours; ABG, arterial blood gases, ARDS, acute respiratory distress syndrome, iNO, inhaled nitric oxide; PaO_2_, partial oxygen pressure in arterial blood; FiO_2_, fraction of inspired oxygen; PaCO2, partial pressure of carbon dioxide; ICU, intensive care unit; #the proportion of prone positioning before iNO initiation was 69.6% (176/253) in patients with PaO_2_/FiO_2_ ratio <150 mmHg and 69.4% (118/170) in patients with PaO_2_/FiO_2_ ratio <100 mmHg.

## Table S3. Oxygenation response to inhaled nitric oxide therapy in patients with COVID-19-related acute respiratory distress syndrome

|  |  | **Pa**O_2_**/Fi**O_2_ | |  |  |
| --- | --- | --- | --- | --- | --- |
| **Time frame** | **N** | **Before iNO**  **initiation** | **After iNO**  **initiation** | **Percent**  **change** | **Response**  **rate** |
| Within 6 hours | 151 | 86 (71-119) | 110 (82-150) | 15.1 (-6.4 - 47.5) | 69 (45.7%) |
| Within 24 hours – best ratio | 246 | 93 (71-120) | 125 (84-172) | 29.5 (-0.7 - 66.1) | 140 (56.9%) |
| Within 48 hours – best ratio | 166 | 95 (69 - 123) | 131 ( 88 - 174) | 27.5 (-6.3 - 89.7) | 91 (54.8%) |
| Within 72 hours – best ratio | 130 | 94 (72 - 122) | 126 (89 - 160) | 25.4 (-6.5 - 66.3) | 70 (53.8%) |
| At least one response during iNO treatment | 256 | 92 (69-120) | 127 (84-177) | 36.5 (8.7-69.1) | 180 (70.3%) |

iNO, inhaled nitric oxide; PaO_2_, partial oxygen pressure in arterial blood; FiO_2_, fraction of inspired oxygen.

##

## Table S4. Change in arterial blood gas variables within six hours of inhaled nitric oxide therapy in patients with COVID-19-related mild, moderate and severe acute respiratory distress syndrome

|  |  |  | **Values within 6 hours of iNO treatment according to ARDS severity at baseline** | | | |
| --- | --- | --- | --- | --- | --- | --- |
|  |  | **N** | **Mild**  **(n=4)** | **Moderate**  **(n=51)** | **Severe**  **(n=95)** | **All**  **(n=150)** |
| FiO_2_ (%) | Absolute change | 150 | -15 (-25 to -5) | 0 (0 to 0) | 0 (0 to 0) | 0 (0 to 0) |
|  | Relative change (%) | 150 | -18.3 (-25 to -8.3) | 0 (0 to 0) | 0 (0 to 0) | 0 (0 to 0) |
| PaO_2_ (mmHg) | Absolute change | 150 | -60.5 (-123 to -3) | 9 (-7.7 to 26) | 15.8 (-1 to 35.5) | 11 (-3 to 31) |
|  | Relative change (%) | 150 | -27.9 (-53.7 to -2.1) | 10 (-6.4 to 32.3) | 25 (-1.7 to 62.4) | 14.7 (-4.9 to 47.1) |
| **Pa**O_2_**/Fi**O_2_ (mmHg) | **Absolute change** | **150** | **-43.8 (-84.8 to**  **18)** | **2.9 (-12.6 to**  **44.4)** | **19 (-4 to**  **56.3)** | **15 (-7.9 to 46)** |
|  | **Relative change (%)** | **150** | **-17.4 (-37.7 to**  **8)** | **2.3 (-9.1 to**  **32.3)** | **30 (-5.3 to**  **64.7)** | **15 (-6.4 to 47.5)** |
| PaCO_2_ (mmHg) | Absolute change | 150 | -5.3 (-15.5 to 2.2) | 0 (-3.0 to 3.5) | 0 (-4 to 4) | 0 (-4 to 3.6) |
|  | Relative change (%) | 150 | -10.6 (-24.7 to 5.6) | 0 (-5.8 to 7.5) | 0 (-8.9 to 8.6) | 0 (-8.7 to 7.6) |
| SaO_2_ (%) | Absolute change | 128 | -1 (-2.5 to -0.5) | 1 (-1 to 2) | 3 (0 to 7) | 2 (-1 to 5) |
|  | Relative change (%) | 128 | -1 (-2.5 to -0.5) | 1 (-1 to 2.2) | 3.3 (0 to 7.8) | 2.1 (-1 to 5.4) |
| HCO3- (mmol/L) | Absolute change | 143 | 1 (-2 to 1) | 0 (-1 to 1) | 0 (-1 to 1) | 0 (-1 to 1) |
|  | Relative change (%) | 143 | 4 (-8.3 to 5) | 0 (-4.8 to 3.6) | 0 (-4.4 to 3.6) | 0 (-4.5 to 3.6) |
| pH | Absolute change | 150 | 0 (0 to 0.1) | 0 (0 to 0) | 0 (0 to 0) | 0 (0 to 0) |
|  | Relative change (%) | 150 | 0.5 (0 to 1.5) | 0 (-0.5 to 0.4) | 0.1 (-0.5 to 0.5) | 0 (-0.5 to 0.5) |
| Methemoglobin (%) | Absolute change | 45 | -0.3 (-0.3 to -0.3) | 0 (-0.2 to 0.4) | 0.1 (0 to 0.3) | 0.1 (-0.1 to 0.3) |
|  | Relative change (%) | 44 | -27.3 (-27.3 to -27.3) | 0 (-28.6 to 50) | 11.3 (0 to 37.5) | 9.5 (-12.7 to 40.2) |
| Lactates (mmol/L) | Absolute change | 126 | 0.1 (0 to 0.2) | 0 (-0.3 to 0.1) | 0 (-0.1 to 0.3) | 0 (-0.2 to 0.2) |
|  | Relative change (%) | 126 | 9.5 (1.6 to 30) | 0 (-18.2 to 12.5) | 0 (-11.1 to 26.7) | 0 (-11.1 to 22.2) |

ARDS, acute respiratory distress syndrome; iNO, inhaled nitric oxide; PaO_2_, partial oxygen pressure in arterial blood; FiO_2_, fraction of inspired oxygen; PaCO2, partial pressure of carbon dioxide; SaO_2_, oxygen saturation in arterial blood. Data are median (interquartile range)
